# Extracellular vesicles isolated from patients undergoing remote ischemic preconditioning decrease hypoxia-evoked apoptosis of cardiomyoblasts after isoflurane but not propofol exposure

**DOI:** 10.1371/journal.pone.0228948

**Published:** 2020-02-14

**Authors:** Frederik Abel, Florian Murke, Morten Gaida, Nicolas Garnier, Crista Ochsenfarth, Carsten Theiss, Matthias Thielmann, Petra Kleinbongard, Bernd Giebel, Jürgen Peters, Ulrich H. Frey

**Affiliations:** 1 Klinik für Anästhesiologie und Intensivmedizin, Universität Duisburg-Essen & Universitätsklinikum Essen, Essen, Germany; 2 Institut für Transfusionsmedizin, Universität Duisburg-Essen & Universitätsklinikum Essen, Essen, Germany; 3 Klinik für Anästhesiologie, Operative Intensivmedizin, Schmerz- und Palliativmedizin, Marien Hospital Herne, Universitätsklinikum der Ruhr-Universität Bochum, Bochum, Germany; 4 Institut für Anatomie, Abteilung für Cytologie, Ruhr-Universität-Bochum, Bochum, Germany; 5 Klinik für Thorax- und Kardiovaskuläre Chirurgie, Universität Duisburg-Essen & Universitätsklinikum Essen, Essen, Germany; 6 Institut für Pathophysiologie, Universität Duisburg-Essen & Universitätsklinikum Essen, Essen, Germany; Indiana University School of Medicine, UNITED STATES

## Abstract

Remote ischemic preconditioning (RIPC) can evoke cardioprotection following ischemia/reperfusion and this may depend on the anesthetic used. We tested whether 1) extracellular vesicles (EVs) isolated from humans undergoing RIPC protect cardiomyoblasts against hypoxia-induced apoptosis and 2) this effect is altered by cardiomyoblast exposure to isoflurane or propofol. EVs were isolated before and 60 min after RIPC or Sham from ten patients undergoing coronary artery bypass graft surgery with isoflurane anesthesia and quantified by Nanoparticle Tracking Analysis. Following EV-treatment for 6 hours under exposure of isoflurane or propofol, rat H9c2 cardiomyoblasts were cultured for 18 hours in normoxic or hypoxic atmospheres. Apoptosis was detected by flow cytometry. Serum nanoparticle concentrations in patients had increased sixty minutes after RIPC compared to Sham (2.5x10^11^±4.9x10^10^ nanoparticles/ml; Sham: 1.2x10^11^±2.0x10^10^; p = 0.04). Hypoxia increased apoptosis of H9c2 cells (hypoxia: 8.4%±0.6; normoxia: 2.5%±0.1; p<0.0001). RIPC-EVs decreased H9c2 cell apoptosis compared to control (apoptotic ratio: 0.83; p = 0.0429) while Sham-EVs showed no protection (apoptotic ratio: 0.97). Prior isoflurane exposure *in vitro* even increased protection (RIPC-EVs/control, apoptotic ratio: 0.79; p = 0.0035; Sham-EVs/control, apoptotic ratio:1.04) while propofol (50μM) abrogated protection by RIPC-EVs (RIPC-EVs/control, Apoptotic ratio: 1.01; Sham-EVs/control, apoptotic ratio: 0.94; p = 0.602). Thus, EVs isolated from patients undergoing RIPC under isoflurane anesthesia protect H9c2 cardiomyoblasts against hypoxia-evoked apoptosis and this effect is abrogated by propofol. This supports a role of human RIPC-generated EVs in cardioprotection and underlines propofol as a possible confounder in RIPC-signaling mediated by EVs.

## Introduction

Remote ischemic preconditioning (RIPC) by repetitive suprasystolic pressure inflations/deflations of a limb blood pressure cuff is an attractive method to decrease perioperative myocardial damage resulting from ischemia/reperfusion (I/R) injury in patients undergoing coronary artery bypass grafting (CABG) [1]. This procedure can decrease cardiac troponin I concentrations and even improve the patients`prognosis [2–3]. However, while the efficacy of RIPC has been proven in various animal studies [4], data from clinical studies are contradictory [2,5–8], but this may be explained by the choice of the anesthetic regimen used. Cardioprotection has been reported in patients receiving the volatile agent isoflurane, but not in those undergoing propofol anesthesia [7–9]. In fact, there is evidence that propofol anesthesia abolishes the protective effects of RIPC [10].

While the precise signal transduction mechanisms of RIPC-evoked cardioprotection in humans are still unknown, humoral factors seem to be involved [11]. Recently, extracellular vesicles (EVs) were hypothesized to participate as humoral mediators of protective signals to the heart to evoke RIPC [12–15].

EVs encompassing exosomes, microvesicles, and apoptotic bodies are nanosized membrane-surrounded structures actively secreted by many cell types and they contain lipids, proteins, mRNAs, and/or micro-RNAs (miRNAs) [16]. Since the EVs content can be incorporated into cells, they are considered novel and complex mediators of intercellular signalling. Accordingly, EVs have become an important focus for physiological and pathophysiological research [17]. In turn, assuming humoral mediation by EVs of the RIPC-evoked cardioprotective signal, propofol might be a confounder inhibiting such an EV-mediated signal.

An increase of EV plasma concentrations, very likely exosomes, following RIPC has been reported in healthy male volunteers [18–19], and we recently showed an increase of EV serum concentrations harboring an altered micro-RNA signature in CABG patients undergoing RIPC [20]. However, it remained unknown whether human serum-derived EVs after RIPC result in cellular protection. We, therefore, assessed whether 1) EVs isolated from RIPC patients evoke protection of cardiomyoblasts (H9c2 cells) against hypoxia-induced apoptosis; and 2) the volatile anesthetic isoflurane and the intravenous anesthetic propofol alter any such effects.

## Methods

### Patient study

Following approval of the local ethics committee (University of Duisburg-Essen, no. 08–3683), written informed consent was obtained from all subjects participating in the trial. The main trial was registered prior to patient enrollment at clinicaltrials.gov (NCT01406678, Principal investigator: Matthias Thielmann, Date of registration: December 1, 2009). 329 patients undergoing elective isolated first-time CABG had been enrolled in a randomized, prospective, double-blind, placebo-controlled study without (Sham) or with RIPC during isoflurane/sufentanil anaesthesia. The study has been performed according to the Declaration of Helsinki and details of the trial and extensive study protocol were published previously [2]. Briefly, anesthesia was induced using etomidate (0.3mg kg^-1^), sufentanil (1μg kg^-1^), and rocuronium (0.6mg kg^-1^) and maintained by isoflurane (end‐tidal concentration: 0.6%‐1.0%) and sufentanil (1‐4μg kg^−1^), as required. In the RIPC group 3 cycles of 5-minute ischemia and 5-minute reperfusion of left upper limb ischemia were evoked after induction of anesthesia by a blood-pressure cuff applied to left upper arm and inflated to >200 mmHg (i.e., at least 15 mmHg higher than the patient’s actual systolic pressure). In the Sham group, the blood-pressure cuff was left deflated for 30 minutes. Blood (10 ml) from patients was obtained from the right radial artery before induction of anesthesia in the awake state and 60 minutes after 3 cycles of left arm ischemia/reperfusion and serum was prepared by letting the blood to clot followed by removal of the clot by centrifugation at 2,000*g* for 10 minutes. Serum was immediately stored at -80°C. Using the serum approach, EVs and other factors which are released from activated platelets are utilized in a standardized fashion thereby reducing an activation of platelets during sample preparation.

In a randomized subprotocol (amendment: 08-21-2011, also approved by the ethics commission), we used a proof-of principle design to prospectively investigate whether the RIPC maneuver results in a changed EV concentration along with the search for humoral factors transferring a protective RIPC signal [21]. For the present *in vitro* study, EVs from 10 randomly selected patients (n = 5 RIPC, n = 5 Sham) matched for similar demographics were used.

### EV isolation and purification

EVs were isolated from serum by using a precipitation solution and centrifugation. Briefly, serum was defrosted and centrifuged at 3,000*g* (5424R Eppendorf, Hamburg, Germany) for 15 minutes at 4°C removing cells and debris. 400μl of supernatant was transferred into DNA LoBind Tubes (Eppendorf, Hamburg, Germany). 200μl of ExoQuick Exosome Precipitation Solution (System Biosciences, Palo Alto, CA) was added and filled up with 400μl 0.9% NaCl/10mM Hepes. Following incubation overnight for 18 hours, the samples were centrifuged at 1,500*g* for 30 minutes at 4°C. Supernatants were removed and EV pellets resuspended in 200μl 0.9% NaCL/10mM Hepes. Finally, the samples were purified by gel filtration using PD SpinTrap G-25 columns (GE Healthcare Europe, Freiburg, Germany) according to the manufacturer’s instructions to remove remaining polyethylene glycol. Again, samples were stored at -80°C until usage.

### Western blotting

For EV Western Blots, the samples were immediately put on ice after RNAse A (Thermo Scientific, Schwerte, Germany) treatment. The resuspended EVs were lysed using RIPA buffer and complete mini protease inhibitors (Roche, Mannheim, Germany). Samples were incubated on ice for 20 minutes with occasional mixing, followed by centrifugation for 20 minutes at 12,000*g* (5424R Eppendorf, Hamburg, Germany) and 4°C. Supernatants were removed to a clean tube and pellets discarded. Protein concentration was measured using the Pierce® BCA Protein Assay kit (Thermo Scientific, Rockford IL) and read using the NanoDrop (Thermo Scientific, Schwerte, Germany) according to the manufacturer’s instructions. Laemmli Buffer with 8% beta mercaptoethanol and 6% sodium dodecyl sulfate (SDS) was used and 30μg from all samples (excluding the samples for CD146 analysis and including HL60 whole cell lysate as a positive control) were applied to a 10% reducing gel. Electrophoresis was carried out using SDS Running buffer (0.01% SDS). Samples to be probed for CD146 were prepared using native loading buffer without SDS and beta mercaptoethanol and run on a 4–20% MP-TGX gel (Bio-Rad Laboratories, Basel, Switzerland) with native running buffer (tris-glycine without SDS). Electrophoretic transfer to nitrocellulose membranes was completed using a semi-dry transfer machine. Membranes were blocked using 5% nonfat dry milk in TBS-Tween (0.1%) (TBS-T) (for flotillin, calnexin and CD146) and 2.5% bovine serum albumin (BSA) in TBS-T (for CD63) for 1 hour at room temperature. Primary antibodies flotillin 1 (C2) (sc-74566) and Mel-CAM (CD146) (sc-18837, Santa Cruz Biotechnology, Heidelberg, Germany) and rabbit anti calnexin (ab133615, Abcam, Cambridge, UK) were diluted in 5% milk/TBS-T 1:200 (flotillin), 1:500 (CD146) and 1:1000 (calnexin). Primary antibody for CD63 was diluted 1:1000 in 0.5% BSA in TBS-T. Membranes were incubated with primary antibody at 4°C overnight. Membranes were then washed 3x10 minutes with TBS-T. Secondary antibodies (goat anti-mouse IgG H&L (HRP) preadsorbed (ab97040, Abcam, Cambridge, UK) and mouse anti-rabbit IgG (HRP) (sc-2357, Santa Cruz Biotechnology, Heidelberg, Germany) were diluted in 5% milk-TBS-T 1:10,000 (mouse) and 1:2000 (rabbit) and incubated at room temperature for 1 hour. Final washing was completed using TBS-T 3x10 minutes. Signal Fire Plus ECL reagent (Cell Signaling, Frankfurt am Main, Germany) was used to visualize the proteins and membranes were visualized using the BioRad ChemiDoc XRS+ Imaging System and the software ImageLab 5.2.1 (BioRad, Basel, Switzerland).

### Quantification and size assessment of EVs

For EV quantification and assessment of nanoparticle size and its distribution, Nanoparticle Tracking Analysis (NTA) was performed (ZetaView, Particle Metrix, Meerbusch, Germany). All samples were diluted 1:5,000, 1:10,000, or 1:20,000 with 0.9% NaCl depending on the sample’s particle concentration, leading to particle concentrations of approx. 5–6x10^7^ ml^-1^. The temperature was maintained at 25°C (±1). Each sample was measured at 11 different positions with 5 cycles of reading at each position. Pre-acquisition parameters were set to a sensitivity of 75, a frame rate of 30 frames per second, and a shutter speed of 75. Post-acquisition parameters were set to a minimum brightness of 20, a maximum size of 200 pixels, and a minimum size of 5 pixels. Only data for particle’s size of 70–150 nm (region of interest, presumed to include exosomes and microvesicles) were considered relevant for data analysis.

### Cell line and culture conditions

H9c2 rat cardiomyoblasts (LGC Standards, Wesel, Germany) were maintained in Dulbecco’s Modified Eagle’s Medium + 25mM Hepes (DMEM GlutaMAX, Thermo Scientific, Schwerte, Germany) supplemented with 10% fetal calf serum (FCS) in a 5% CO_2_ atmosphere at 37°C. Subconfluent cells (70–80%) were split at 1:3 or 1:4 ratio and used until passage 25.

### Labeling of EVs and assessment of EV uptake in H9c2 cells

Labeling procedures were carried out in accordance to a standard labeling protocol with modifications (Invitrogen, Carlsbad, USA). Briefly, H9c2 cells were incubated overnight on 8-well chamber slides in standard medium (DMEM with 10% FCS; 1.6x10^4^ cells/200μl). Isolated EVs were labeled with 1μl BODIPY TR Ceramide stock solution (Invitrogen, Carlsbad, USA) per 100μl biological sample in PBS for 20 minutes at 37°C. Excess Bodipy was removed using Exosome spin columns (Thermo Scientific, Schwerte, Germany). H9c2 cells were incubated with labeled EVs at three different EV-concentrations (1x10^5^, 1x10^7^, and 1x10^9^ nanoparticles/ml) for 1, 3, 6, and 18 hours in DMEM with 10% FCS. After incubation, the medium was removed and cells were washed with PBS followed by a 20-minute fixation period at room temperature using 4% paraformaldehyde (PFA) in PBS. Permeabilization was done using 0.1% Triton X-100 in PBS for 3 minutes at room temperature. Cells were then washed with PBS. F-Actin cytoskeleton subunits were labeled with green Alexa Fluor 488 Phalloidin (Life Technologies Corporation, Carlsbad, USA) for 20 minutes. Samples were mounted with ProLong Gold Antifade Mountant with DAPI (Invitrogen, Carlsbad, USA) for DNA labeling and coverslips for 24 hours. All slides were analyzed using an Axio Observer.Z1 fluorescence microscope and Zeiss Zen Software (Carl Zeiss AG, Oberkochen, Germany).

**Fig 1:**
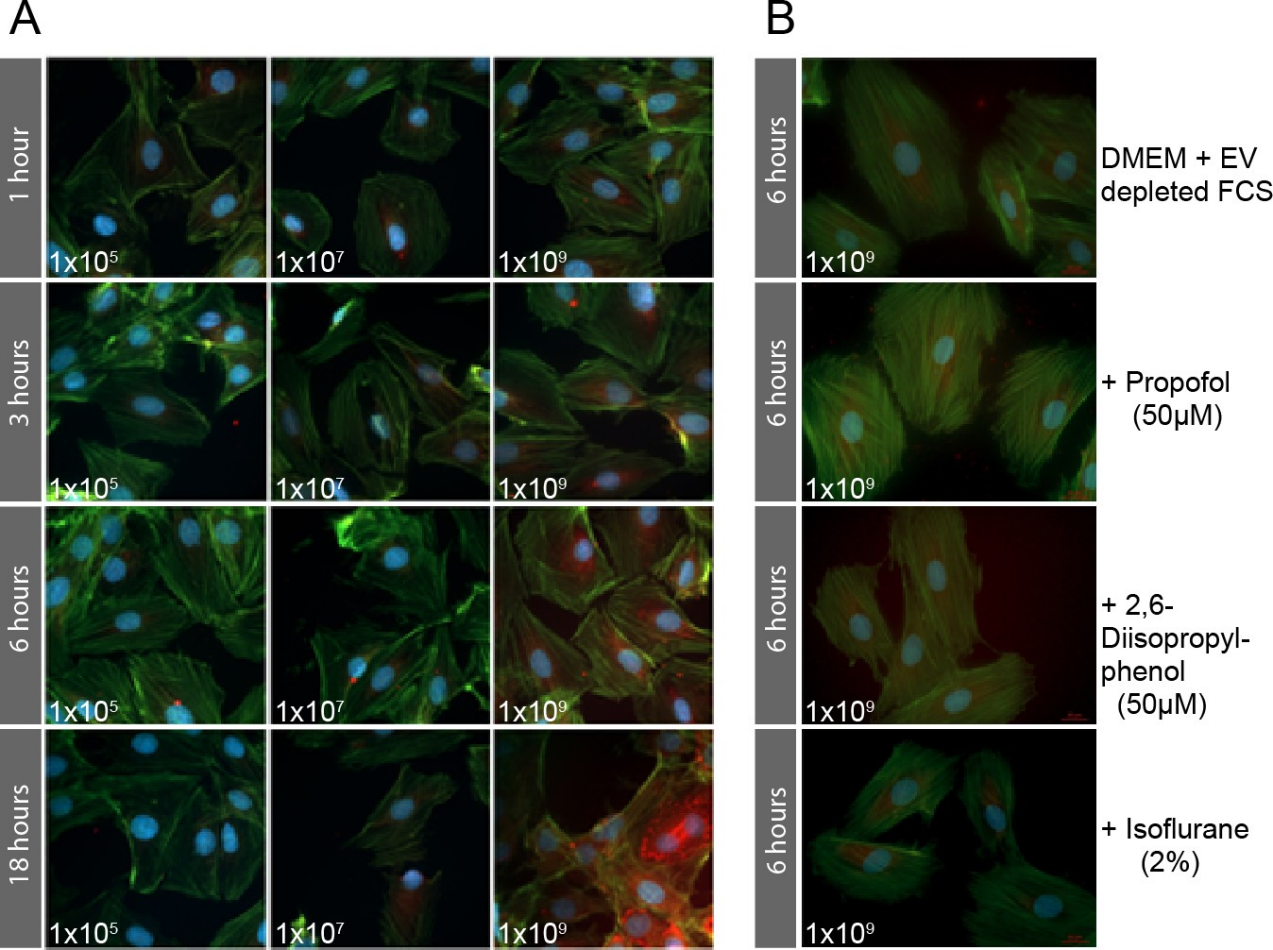
A. Intracellular uptake of BODIPY TR ceramide labeled EVs (red) into H9c2 cells. Association of EV concentration and incubation time in H9c2 cells as assessed by fluorescence microscope. Strongest fluorescence signals of intracellular EVs (red dots) were seen at an EV concentration of 1x10^9^ nanoparticles/ml with incubation periods of 1, 3, 6, and 18 hours with increased signaling intensity over time. Lesser concentrations of 1x10^5^ and 1x10^7^ part/ml did not evoke a detectable increase in fluorescence after any incubation time. Accordingly, a combination of 1x10^9^ nanoparticles/ml and 6 hours incubation time was used for further experiments to allow for adequate cellular EV uptake while avoiding potential oversaturation. B. Uptake of labeled EVs into H9c2 cells cultured with EV-depleted FCS Taking advantage of prior results, the experiment was repeated using the optimum incubation time and concentration for EV-uptake with DMEM and EV-depleted FCS (DMEM + FCS–EV) including four different setups: DMEM + FCS–EV, propofol soya emulsion in DMEM + FCS–EV, pure 2,6-diisopropylphenol in DMEM + FCS–EV, and isoflurane. As expected, the overall signal of labeled EVs in the cells was lower using EV-depleted FCS in compared to the first set of experiments with EV containing FCS. The cells show a similar EV-uptake in all conditions.

Uptake kinetics of visualized EVs into H9c2 cells depended both on EV concentration and duration of incubation. While the strongest fluorescence signals of intracellular EVs were detected at a concentration of 1x10^9^ nanoparticles/ml for all incubation periods (Fig 1A), the combination of 1x10^9^ nanoparticles/ml and 6 hours incubation time was used for further experiments to allow for adequate cellular EV uptake without the risk of saturation effects as observed with 18 hours of incubation (Fig 1A).

For the second set of uptake experiments we cultured the H9C2 cells in DMEM with 10% exosome depleted FCS (DMEM + FCS–EV) (Exosome-depleted Fetal Bovine Serum Qualified One Shot^™^, Gibco, Life Technologies, Carlsbad, USA). Using the optimum time and concentration determined in the first set of experiments we then tested four different conditions; DMEM + FCS–EV, 50μM propofol soya emulsion (Propofol Claris MCT, Pharmore GmbH, Ibbenbüren, Germany) in DMEM + FCS–EV, 50μM pure 2,6-diisopropylphenol (Sigma Aldrich, Darmstadt, Germany) in DMEM + FCS–EV, and 2% isofluran (Isofluran Baxter, Unterschließheim, Germany) (Fig 1B). EV labeling as well as fixation, actin and DAPI labeling was completed as described above. All slides were analyzed using an Axio Observer.Z1 fluorescence microscope and Zeiss Zen Software (Carl Zeiss AG, Oberkochen, Germany).

### Experimental setup, normoxic/hypoxic treatment

**Fig 2:**
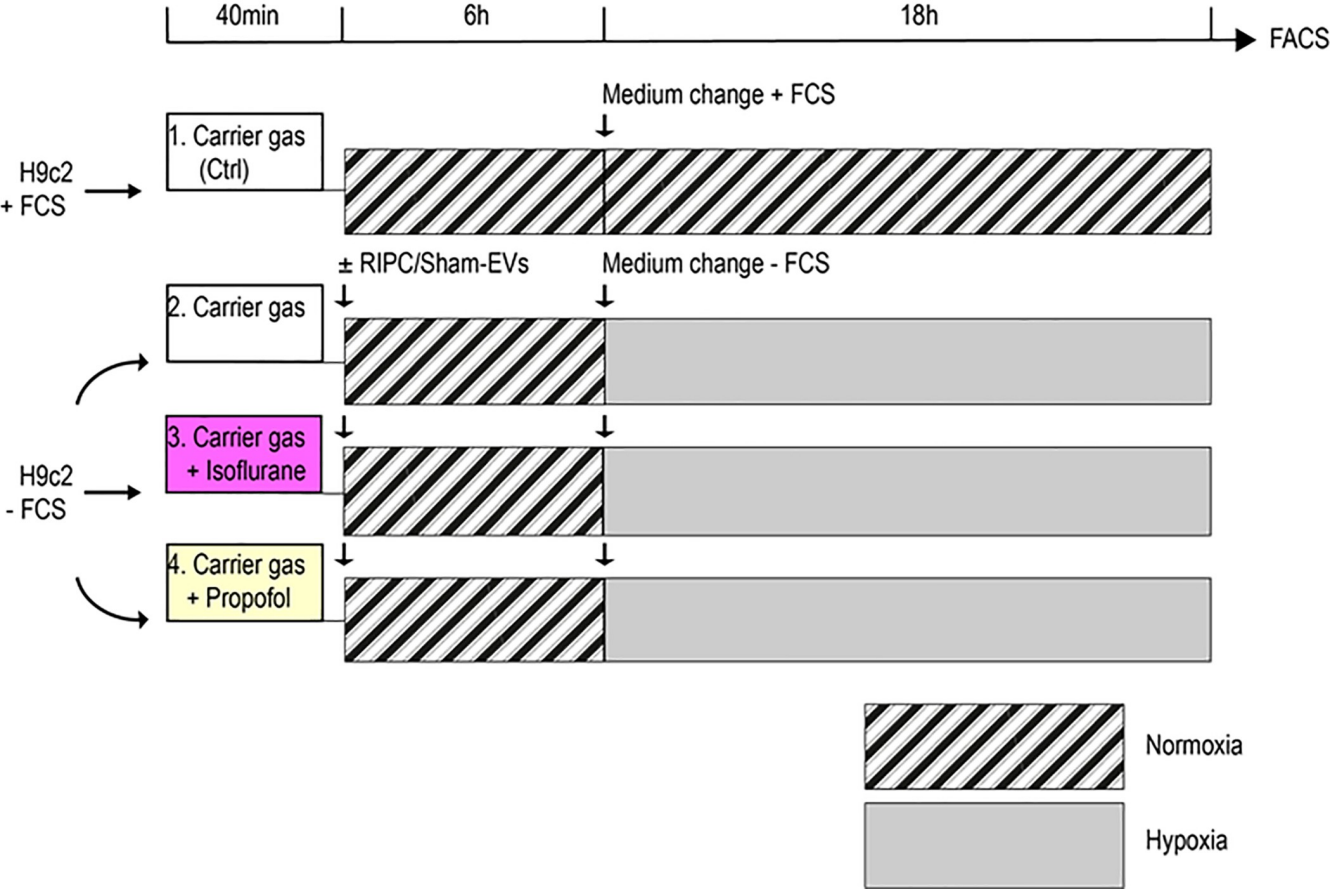
Experimental setup of H9c2 cells exposed to normoxia/hypoxia. Each line represents a different condition. Cells were transferred to 6-well plates and incubated for 24 hours in standard DMEM + 25mM Hepes. The medium was then changed to fresh DMEM + 25mM Hepes ± FCS for each condition. All plates were perfused with normoxic gas (21% O_2_, 5% CO_2_), whereas one plate was additionally exposed to isoflurane 2% (magenta filled box) and one plate to 50μM propofol (yellow filled box). After 40 minutes EVs were added at a concentration of 1x10^9^ nanoparticles/ml. Normoxia treated cells did not receive EVs. After incubation for 6 hours, the medium was renewed again to DMEM + FCS in cells under normoxic conditions or to DMEM—FCS in cells undergoing hypoxia. H9c2 cells were then cultured in a normoxic or hypoxic atmosphere (1% O_2_, 5% CO_2_) for 18 hours. Apoptosis under all experimental conditions was measured by flow cytometry as the endpoint. The hatched boxes illustrate normoxic periods and the grey filled boxes hypoxic periods.

H9c2 cells were kept in either normoxic and hypoxic atmospheres under exposure of isoflurane or propofol according to a model described previously [22]. Twenty-four hours before the experiment, cells had been seeded with 7.5x10^4^ cells/well in 6-well-plates. Cells were allocated to 4 different general conditions: (1) normoxia (control), (2) hypoxia, (3) hypoxia + isoflurane, and (4) hypoxia + propofol (Fig 2).

For each condition, medium was renewed (DMEM + 25mM Hepes ± 10% FCS). Fetal calf serum containing medium was used for normoxic and FCS-depleted medium for hypoxic treated cells. Cells were transferred into Billup-Rothenburg chambers (MIC-101, Billups-Rothenburg, Del Mar, CA) following flushing the chamber (5l/min for 40 minutes) with a normoxic carrier gas (5% CO_2_, 21% O_2_, balance nitrogen; Air Liquide, Düsseldorf, Germany). Isoflurane (2%) was delivered to the normoxic gas flow using an in-line calibrated isoflurane vaporizer (Dräger, Lübeck, Germany). Exhaust isoflurane concentration was monitored by an anesthetic agent analyzer (Dräger Vamos, Lübeck, Germany). Propofol (50μM) soya oil emulsion was supplemented to the cells’ medium. EVs isolated from RIPC patients before and 60 minutes after the RIPC maneuver as well as EVs from Sham patients were added to cells over a period of 6 hours before undergoing hypoxic treatments whereas cells under normoxic conditions did not receive EVs. After EV incubation, the medium was renewed again and the chambers were flushed with either normoxic and hypoxic (5% CO_2_, 1% O_2_) atmospheres and cells were cultured for another 18 hours.

The EV concentration was normalized to 1x10^9^ nanoparticles/ml, resulting in sample volumes between 2–11μl. Control cells without EV incubation always received a matched volume of NaCl/Hepes.

Temperature was kept at 37°C. The endpoint was apoptosis as detected by flow cytometry. Experiments were performed at least five times for each condition.

### Flow cytometry

After the experimental procedures, cells were collected and resuspended in 100μl of 1x Annexin binding buffer. Subsequently, cells were stained with 5μl Annexin V-FITC (BD Biosciences, Heidelberg, Germany) and 5μl 7-AAD (7-Aminoactinomycin D) (Beckman Coulter, Krefeld, Germany) and incubated for 15 minutes in the dark at room temperature. Every sample was washed with binding buffer, following centrifugation at 900*g* (5424R Eppendorf, Hamburg, Germany) for 5 minutes at 4°C. The pellets were resuspended with 200μl binding buffer and the percentage of apoptotic cells quantified by flow cytometry (Cytoflex S and CytExpert V2.0 software, Beckman Coulter, Krefeld, Germany).

### Statistical analysis

Data are presented as means ± standard error of the mean (SEM) unless stated otherwise. Comparison variables between conditions were analyzed by unpaired or one-sample two-tailed Student t-tests, as indicated after confirmation of normality using the Shapiro-Wilk normality test. Otherwise, the Mann-Whitney U test was used. Statistical analyses were performed by Graph Pad Prism 6 software (Graph Pad Software, La Jolla, CA). An a priori alpha error p less than 0.05 was considered to indicate statistical significance.

## Results

### RIPC increases serum particle concentration in CABG-patients

No significant differences were detected between RIPC and Sham patients regarding demographics and hemodynamics (Table 1).

**Table 1:** Perioperative characteristics of CABG patients undergoing RIPC or Sham under isoflurane / sufentanil anesthesia.

	All	RIPC	Sham	P
n (%)	10	5 (50)	5 (50)	
Age (years)	69.2±2.4	72.4±2.3	66.0±4.0	0.20
Sex (male/female)	9/1	4/1	5/0	
Body mass index (kg/m^-2^)	28.7±2.0	31.6±3.4	25.8±1.6	0.16
Smoking, n (%)	4 (40)	1 (20)	3 (60)	0.52
Preoperative creatinine serum concentration (mg/dl)	1.12±0.04	1.11±0.09	1.13±0.04	0.83
Left ventricular ejection fraction (%)	56±2.5	53±4.36	59±2.26	0.30
**Medication**				
ASS	10 (100)	5 (100)	5 (100)	
Clopidogrel	3 (30)	1 (20)	2 (40)	
β-Blockers	9 (90)	4 (80)	5 (100)	
Statins	10 (100)	5 (100)	5 (100)	
ACEI/ARB^1^	5 (50)	3 (60)	2 (40)	
**Intraoperative characteristics**				
Mean pulmonary artery pressure (mmHg)	21.8±1.4	21.2±1.16	22.4±2.62	0.69
Pulmonary capillary wedge pressure (mmHg)	11.9±1.35	10.6±1.4	13.2±2.3	0.36

Data are presented as means ± SEM or numbers (%).

^1^ACEI: angiotensin converting enzyme inhibitor, ARB: angiotensin receptor blocker

**Fig 3:**
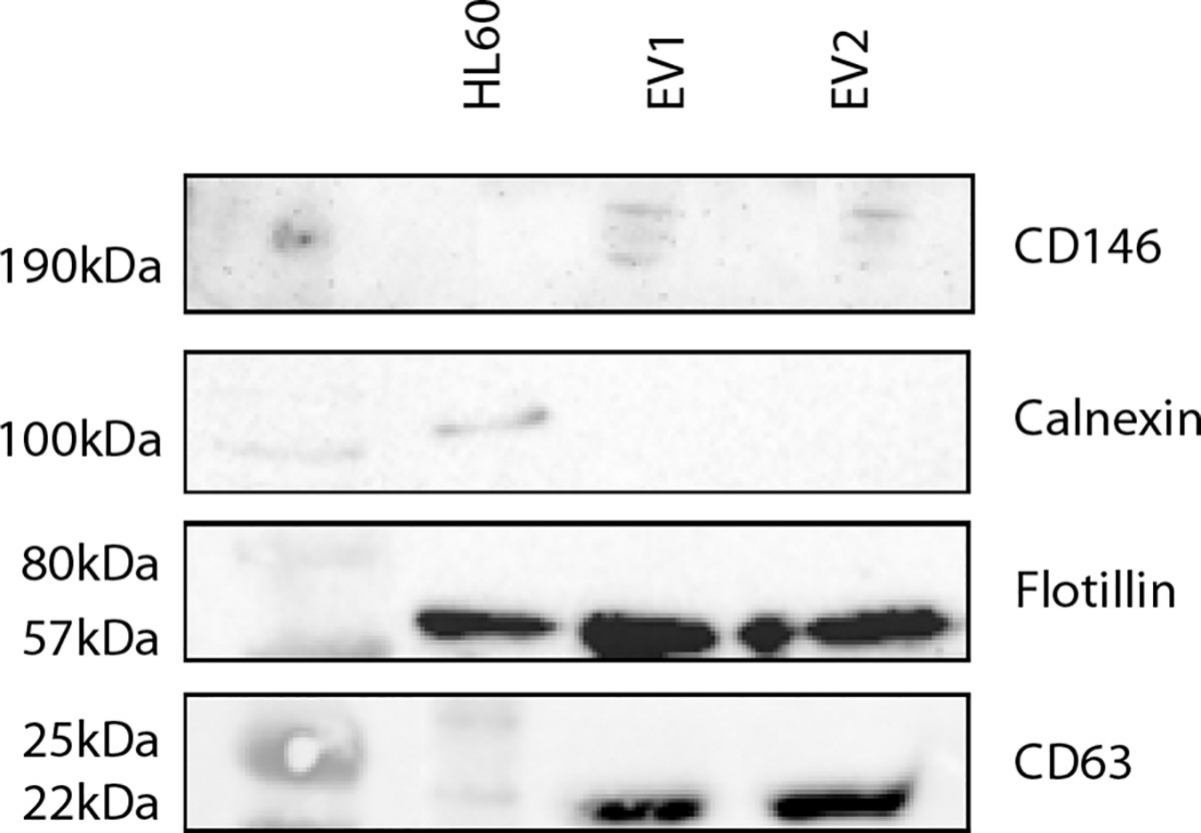
Western blots of EVs isolated from humans undergoing RIPC. Presence of the cytosolic protein flotillin 1, EV marker CD63, endothelial cell marker CD146, and absence of calnexin was analyzed in two representative EV samples extracted of CABG-patients 60 min post-RIPC. HL60 cell lysate was used as a positive control.

Following EV extraction, the EV presence was confirmed by Western blots using flotillin 1 as cytosolic marker protein, CD63 as a vesicle membrane marker protein, and calnexin as a negative control for cell contamination. Furthermore, expression of CD146 was tested to be positive as a marker for endothelial cells. Evidenced by the Western Blots, EVs were successfully isolated and highly enriched within the samples (Fig 3). Whole cell lysate derived from the HL60 cell line (Abcam, Cambridge, UK) was used as positive control.

**Fig 4:**
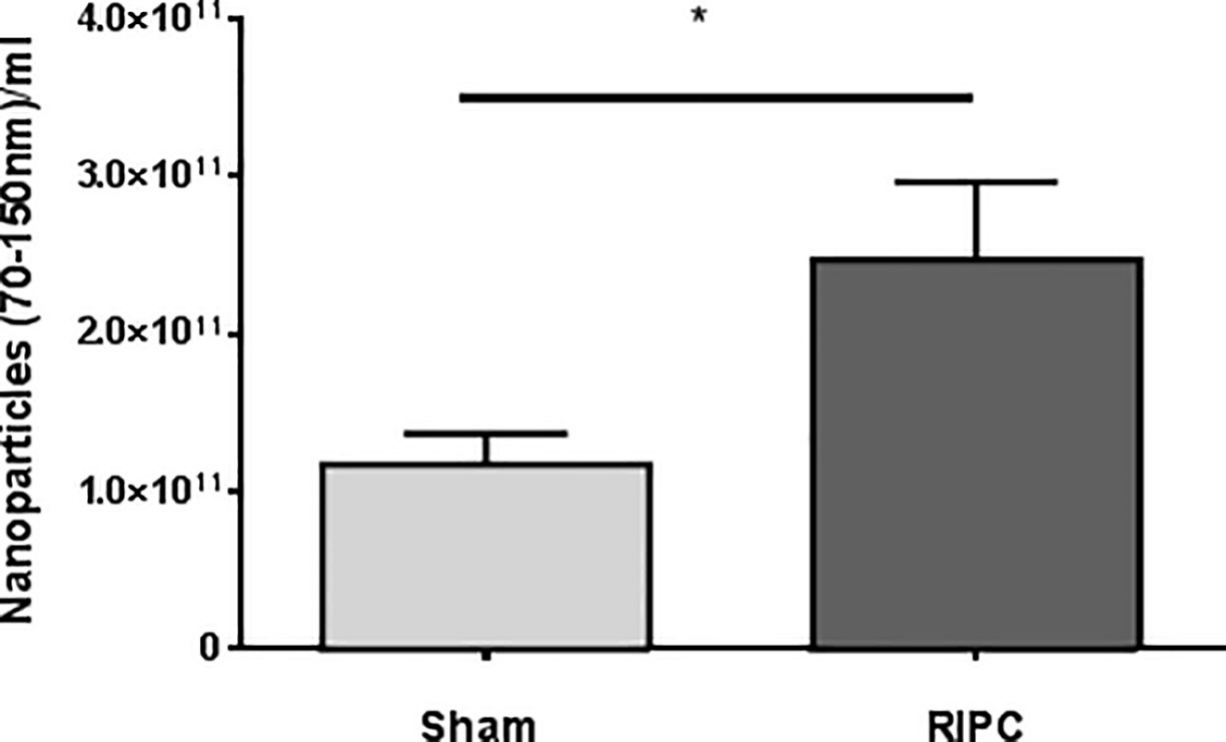
Arterial nanoparticle concentration of patients after RIPC or Sham interventions as analyzed by Nanoparticle Tracking Analysis (NTA). Total serum EV-concentration between 70-150nm/ml obtained from patients 60 minutes after RIPC or Sham treatment. Values are means ± SEM; n = 5 (*p<0.05).

The EV diameter ranged between 70 and 180nm, with an average of 120nm. While baseline EV concentrations in the awake state were not different between RIPC and Sham patients, particle concentrations were twice as high 60 minutes after RIPC (RIPC: 2.5x10^11^±4.9x10^10^ nanoparticles/ml, Sham: 1.2x10^11^±2.0x10^10^ nanoparticles/ml; p = 0.04), suggesting that the RIPC maneuver increased the nanoparticle secretion (Fig 4).

### Hypoxia induced apoptosis in H9c2 cells incubated with or without isoflurane or propofol

**Fig 5:**
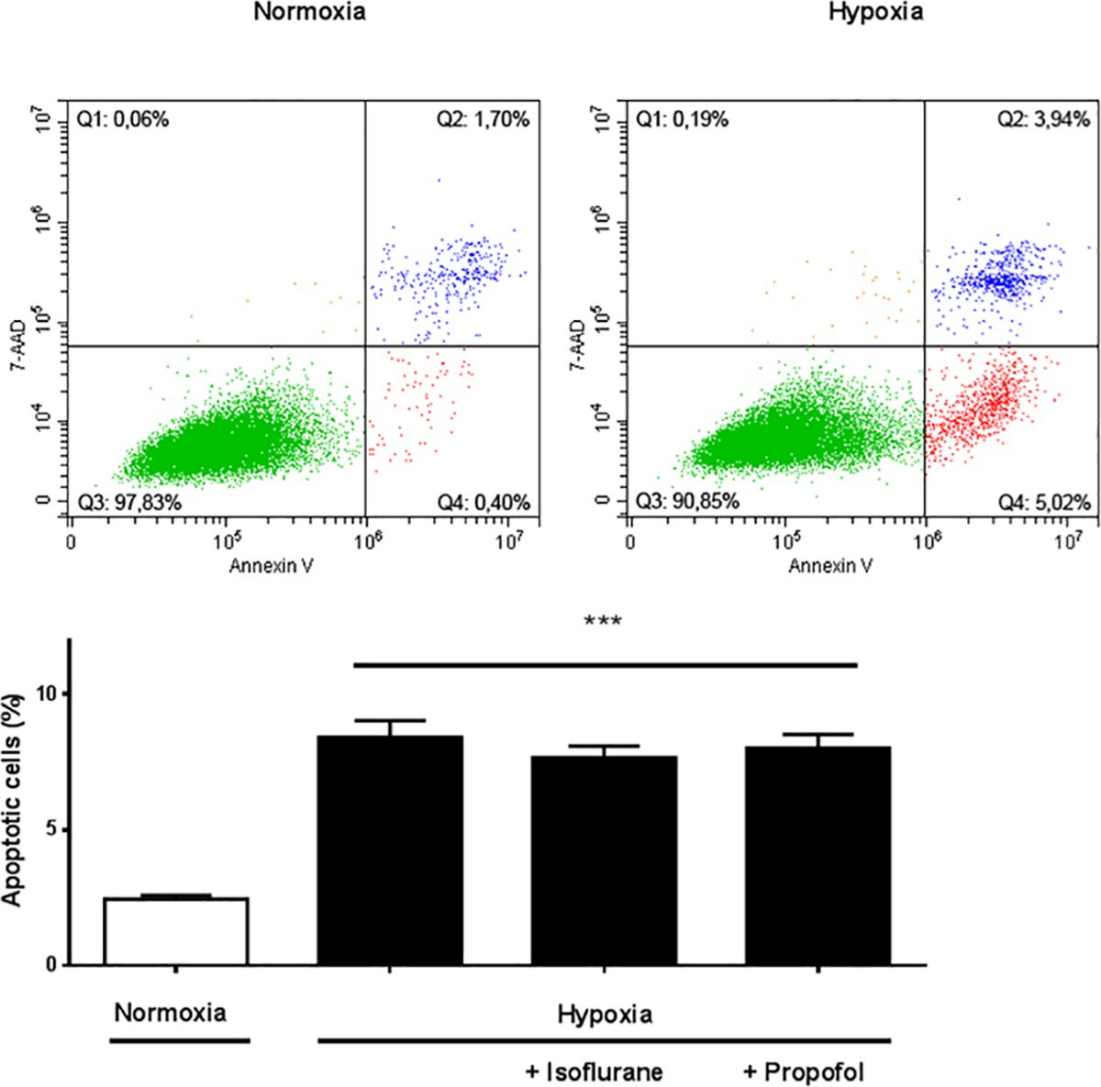
A effect of normoxia/hypoxia exposure on apoptosis rate of H9c2 cells. Representative flow cytometric images after 18 hours of normoxia or hypoxia without incubation with EVs. Apoptosis was detected by Annexin V/7-AAD staining. b Quantitative analysis of apoptotic cells after normoxia/hypoxia and isoflurane or propofol exposure. Apoptosis was markedly increased by hypoxia, whereas isoflurane or propofol had no significant additional beneficial effect. Results are expressed as means ± SEM; n = 10 (***p<0.0001).

Following hypoxia, the presence of apoptotic cells increased to 8.4% compared to 2.5% with normoxia (p<0.0001) (Fig 5A). Cells cultured with hypoxia alone and those exposed to hypoxia plus either isoflurane or propofol showed a similar apoptotic rate, indicating that isoflurane or propofol *per se* neither had a protective nor a damaging effect on cells during hypoxia. (Fig 5B).

### Protection by RIPC-related EV fractions against hypoxia-evoked apoptosis and effects of isoflurane or propofol

**Fig 6:**
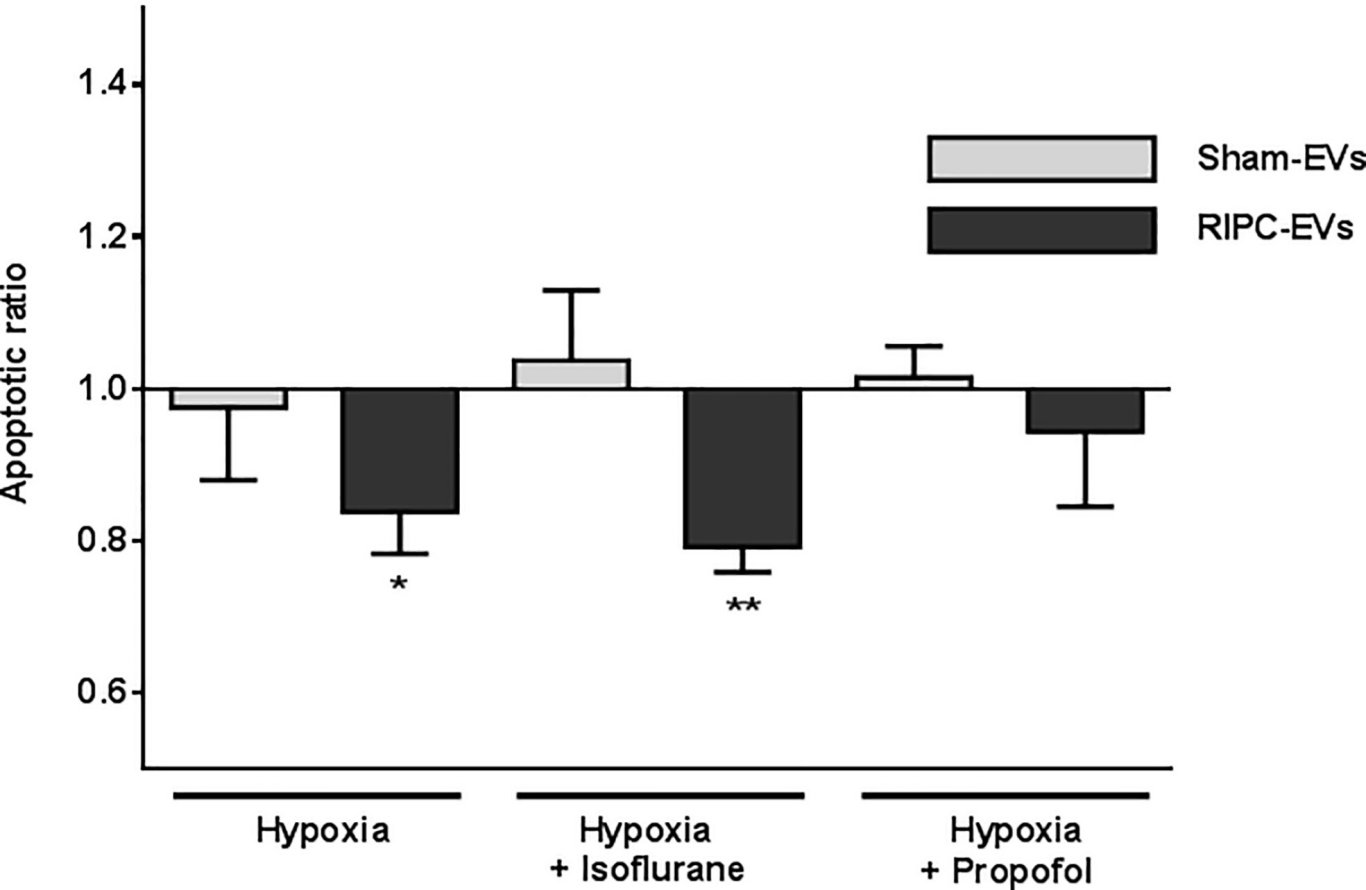
Influence of RIPC-EV or Sham-EV fractions on apoptosis rate in H9c2 cells. EVs were added to H9c2 cells at a concentration of 1x10^9^ nanoparticles/ml and incubated for 6 hours before a change of medium and for the following 18 hours of hypoxia. Apoptotic ratio of cells after hypoxia ± isoflurane or ± propofol plus EVs are presented compared to cells without EV incubation. Values are means ± SEM; n = 5 (*p<0.05, **p<0.01).

We next investigated the effect of the EV fractions isolated from CABG patients following RIPC or Sham treatments with and without isoflurane or propofol on H9c2 cell apoptosis. Results are displayed as apoptotic ratio relative to cells without EV coincubation (Fig 6). Apoptotic ratio significantly decreased in hypoxic cells cultured with RIPC-EVs fractions (apoptotic ratio RIPC-EVs: 0.83±0,06; p = 0.0429; Fig 6). Apoptosis of cells additionally exposed to isoflurane was even lower (apoptotic ratio RIPC-EVs: 0.79±0.03; p = 0.0035). In contrast, apoptosis of H9c2 cells treated with Sham-EV fractions was unaltered compared to cells without EV incubation (apoptotic ratio Sham-EVs: 0.97±0.09; p = 0.81; Sham-EVs + isoflurane: 1.04±0.09; p = 0.71; Fig 6).

Interestingly, apoptotic ratio did not change when cells were incubated with RIPC-EV fractions in the presence of propofol (apoptotic ratio 0.94±0.09; p = 0.602; Fig 6), implying an inhibition by propofol of RIPC-EV-mediated protection. EV fractions from Sham patients also did not affect apoptosis (apoptotic ratio 1.01±0.04).

**Fig 7:**
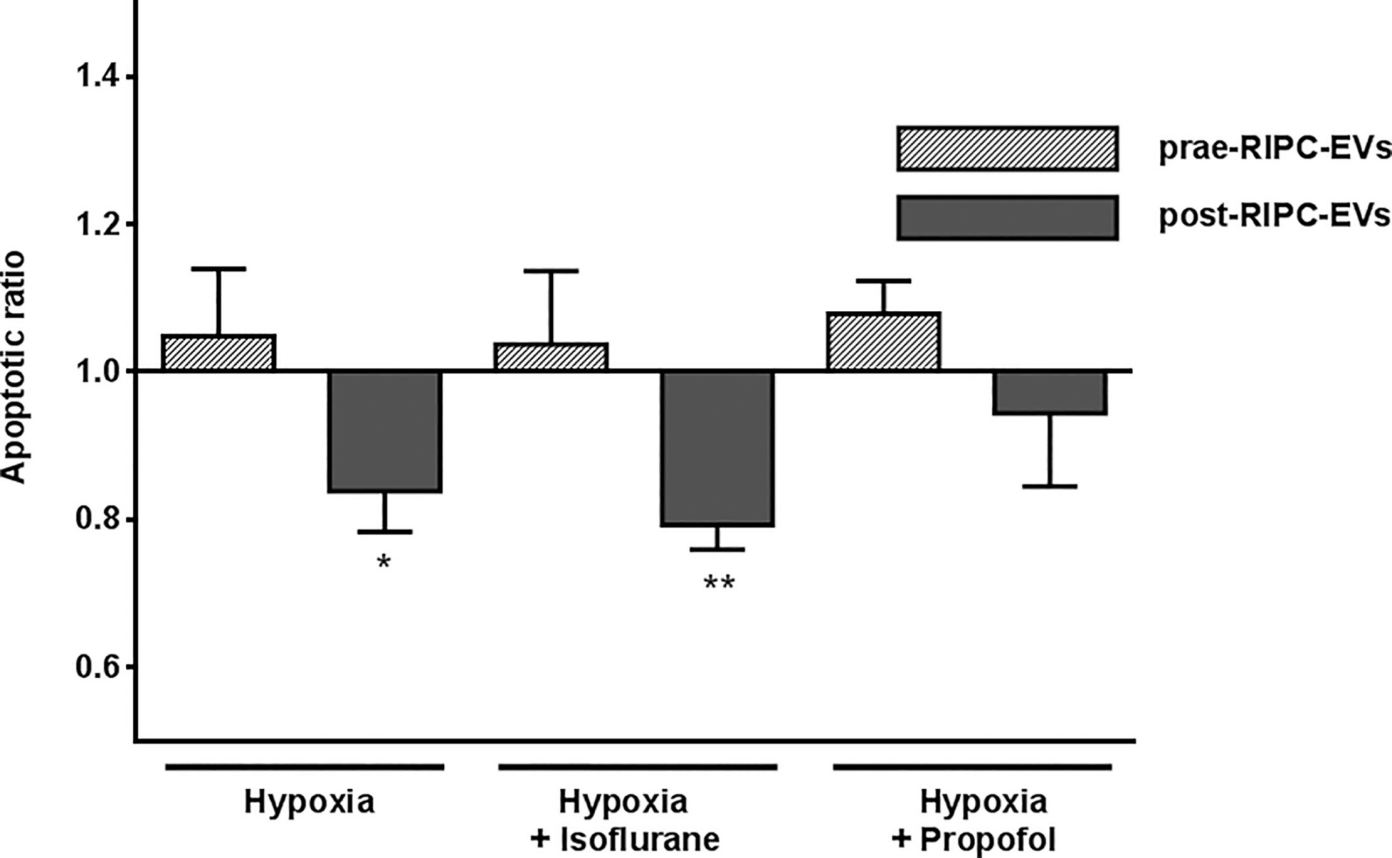
Impact of EV fractions on cell apoptosis isolated before RIPC in contrast to EVs isolated after RIPC. Patient-derived EVs extracted before the RIPC maneuver (prae-RIPC-EVs) had no effect on apoptosis of H9c2 cells compared to cells incubated without EVs. In contrast to EV fractions isolated before the RIPC maneuver, post-RIPC EV fractions improved the apoptotic ratio of H9c2 cells after both hypoxia and hypoxia plus isoflurane. Data are presented as apoptotic ratio of cells with EV-coincubation compared to apoptosis without EV-coincubation. Values are means ± SEM; n = 5 (*p<0.05 **p<0.01).

Finally, to test whether the protective cellular effect is indeed associated with the RIPC maneuver, we used EV fractions obtained from the same patients in the awake state before anesthesia and before the RIPC-maneuver. EV fractions from RIPC-patients but isolated prior to RIPC did not significantly alter apoptotic ratio either after hypoxia alone or when accompanied by isoflurane or propofol when compared to cells without EV incubation (apoptotic ratio prae-RIPC-EVs hypoxia: 1.05±0.09; p = 0.626; hypoxia + isoflurane: 1.04±0.1; p = 0.728; hypoxia + propofol: 1.08±0.04; p = 0.154) supporting the protective effect on cell viability of EV fractions only obtained after RIPC (Fig 7).

## Discussion

EVs have recently attracted attention as mediators of intercellular communication, physiological and pathophysiological effects, and even future carriers of treatment modalities [17]. The cardioprotective potential of human-derived EVs on heart cells remains unclear. We, therefore, assessed whether 1) EV fractions isolated from patients having undergone RIPC evoke protection of cardiomyoblasts (H9c2 cells) against hypoxia-induced apoptosis *in vitro*; and 2) the volatile anesthetic isoflurane and the intravenous anesthetic propofol alter any such effects.

Various humoral and neuronal signal mechanisms as well as neurohumoral interactions have been assumed to mediate the remote ischemic preconditioning signal [23]. Giricz et al. suggested for the first time a potential relevance of EVs for cardioprotection, since transfer of coronary perfusate from a preconditioned rat heart to a recipient rat heart decreased infarct size following 30 min of global ischemia and this protective effect was abolished by EV depletion of the perfusate [13]. Moreover, EV concentrations increased after RIPC performed on hind limbs of rats or forearms of healthy male volunteers [18]. However, while these EVs reduced infarct size when transferred to isolated rat hearts rendered ischemic, cardioprotection was achieved no matter whether these EVs were extracted after RIPC or without RIPC treatment. Finally, larger CD54^+^ and CD146^+^ EV concentrations, considered microvesicles (MVs), originating from endothelial cells were reported to be increased by RIPC in experiments conducted on limbs of healthy humans and rats [19]. In contrast to our findings, however, these larger EVs failed to reduce infarct size when injected intravenously into rats undergoing myocardial ischemia, indicating different cardioprotective efficacies between larger and smaller EV fractions released by RIPC.

In our study, we could show that RIPC increases arterial EV nanoparticle concentrations in humans. Adding these EV fractions to cultured rat H9c2 cells resulted in substantial protection against hypoxia-induced apoptosis, whereas EV fractions from Sham patients in similar particle concentrations did not.

This suggests that RIPC not only evokes increased EV concentrations in the blood but might also lead to a modification of these EVs`cargo and/or surface proteins. This hypothesis is further supported by the fact that EV fractions isolated from RIPC patients had a protective effect only after the RIPC maneuver had been performed, whereas EV fractions obtained both from Sham patients and from RIPC patients but obtained prior to the RIPC maneuver did not decrease apoptosis. Moreover, neither isoflurane nor propofol had an influence on uptake of EVs into H9c2 cells or apoptosis of those in our experiments, suggesting that the different composition of EVs is determining the protective abilities.

Considering the results of previous studies regarding protective properties of post-RIPC EVs, our work adds to these findings by using human-derived EVs and demonstrating for the first time that these EVs evoke cellular protection as well.

It has been demonstrated that circulating EVs possess cardioprotective properties. However, the cellular source of the released EVs by RIPC was not investigated in most studies and thus remains unclear. Most EVs are suggested to originate predominantly from platelets and erythrocytes, but lymphocytes, cardiomyocytes, and other parenchymal cells may contribute as well [15]. Mesenchymal stem cell (MSC)-derived EVs and cardiac progenitor cell (CPC)-derived EVs have also been reported to decrease infarct size and improve cardiac function in experimental studies [24–25]. Endothelial cells, an omnipresent cell type, may also represent a possible source of EVs released by the RIPC maneuver [19]. Ischemic preconditioning results in increased EV production by endothelial cells that are protective against I/R injury in cardiomyocytes via activation of the ERK1/2 MAPK signalling pathway [26–27]. The presence of CD146 as widely used marker for endothelial cells was confirmed in our human EV-samples. It is conceivable that RIPC induces endothelial derived EVs, which are released into the vascular system in consequence of repetitive applied I/R cycles.

Since EVs contain various miRNAs and some miRNAs like miRNA-21, miRNA-24, and miRNA-144 can have cardioprotective effects [28–31], the EV related effects may be mediated by miRNAs. Specifically, miRNA-21 transported within EVs is a good candidate since its expression is upregulated in oxidative stress-induced EVs and EVs from endometrium-derived mesenchymal stem cell (EnMSCs) and miRNA-21 decrease myocardial infarct size in rats as well as apoptosis of cardiomyocytes [28,29,32]. Recently, exosomal miRNA-21a-p5 has been shown to be a cardioprotective factor produced by exosomes and released by mesenchymal stem cells that were cultured with cardiomyocytes before ischemia/reperfusion [33]. These findings are strengthened by our previous work, observing an altered EV miRNA signature, especially miRNA-21, in CABG patients having undergone a RIPC procedure [20].

RIPC also elicits a greater expression of miRNA-24 harbored within EVs and evokes anti-apoptotic effects in H_2_0_2_-treated H9c2 cells following pre-incubation with RIPC-EVs [30]. Finally, cardioprotection by RIPC was associated with increased myocardial miRNA-144 expression, whereas antisense oligonucleotides against miRNA-144 abolished cardioprotection [31].

Therefore, one may speculate that RIPC not only increases arterial EV concentrations, but also alters their cargo increasing their content of cardioprotectively acting miRNAs, which are then transported via the blood stream and taken up by the myocardium and perhaps other parenchymal organs. Nevertheless, besides these considerations on vesicular transfer, many other mechanisms are currently discussed to mediate cardioprotection by RIPC [11].

Interestingly, the cell protective effect by EV fractions obtained after RIPC was abolished by propofol. This observation is in accordance with previous findings demonstrating neutral effects of RIPC in cardiac surgery patients undergoing propofol anesthesia and decreased postoperative troponin concentrations following RIPC in patients under isoflurane but not propofol anesthesia [2,5–7], suggesting an interference effect of propofol with RIPC evoked cardioprotection [9]. Accordingly, that propofol abolished the cellular protection evoked by EV fractions obtained following RIPC in our *in vitro* studies supports these clinical observations. Given the absence of cardioprotection with propofol, it has not yet been addressed at what level this interaction might be located. Propofol inhibits activation of signal transducer and activator of transcription 5 in RIPC patients [8], which implies an interaction at the cellular level and our results confirm this by showing an inhibition by propofol of protection by RIPC-EV fractions on a cellular level. A recent study, however, has pointed out that a loss of RIPC-induced cardioprotection during propofol anaesthesia either depends on inhibiting release of protective humoral factors or the transport of such factors to the myocardium but does not prevent the respective intracellular signalling. Plasma of RIPC-treated rats under pentobarbital anesthesia reduces infarct size when transferred to native rat hearts before global ischemia, whereby such protection is abolished under propofol anaesthesia [34]. Furthermore, it is yet to be clarified whether it is the propofol molecule *per se* or the vehicle fat emulsion that influences EVs, their intracellular entry or composition, or a downstream intracellular effect. One explanation could be an interference of the soya oil emulsion with EV transport or the EVs’ lipid-membranes. Deng et al. observed that propofol without fat emulsion inhibits the release of larger EVs from endothelial cells during hypoxia/reoxygenation supporting that propofol itself represents the inhibitory factor [35]. Since our patients were not anesthetized with propofol, we cannot assess the impact of propofol on serum EV concentrations. In order to give an impetus for such studies, we tested the effect of propofol soya emulsion and the pure agent 2,6-diisopropylphenol without soya emulsion on EV-uptake into H9c2 cells. Both, pure propofol and propofol soya emulsion had no influence on uptake, making a negative interference with intracellular entry of EVs more unlikely. We used a propofol concentration of 50μM to analyse the interference with RIPC-EVs, taking into account that this concentration is applicable for clinical considerations and ranges within the clinically relevant propofol concentrations of 17–62μM. This concentration is also commonly used to investigate the impact of propofol on H9c2 cells undergoing hypoxia/reoxygenation [36–37].

From a therapeutic point of view. it is interesting whether EV fractions isolated from volunteers after RIPC maneuvers or made artificially might confer organ protection in various clinical settings when administered in sufficient dosages systemically, into coronary arteries prior to interventions, or into the aortic root before aortic crossclamping and heart surgery.

### Limitations

For *in vitro* experiments we used immortalized H9c2 cells. While these cells are not human cardiomyocytes, we focused in a first step on hypoxia experiments to prove that human EVs exhibit specific properties resulting in altered apoptosis in these cells. Immortalized neonatal/adult cells like H9c2 or HL-1 cells have been used in many investigations as a surrogate for human cardiomyocytes [38–39] to analyse ischemia/reperfusion injury [40–41]. Furthermore, ischemic states are often simulated in these cells by serum deprivation and hypoxic periods [42–44]. Nevertheless, the characterization of function of human EVs on human clinically relevant samples (e.g., heart and brain) is of major importance for associated future experiments but also raises ethical questions.

While our data show that EV nanoparticle concentrations increase following RIPC and that these EV fractions act in a protective fashion in cells exposed to hypoxia, our present study does not pinpoint the molecular means by which such EVs mediate the protective effect. Furthermore, the role of propofol on EV release was not addressed in our present work and has to be clarified in future studies. Finally, although we used EV fractions isolated from human patients, experiments were conducted *in vitro* with immortalized cells. The protective abilities of EVs from RIPC patients may not directly apply to the human myocardium. In addition, our methods of isolating EVs by precipitation may not have resulted in a pure, homogenous EV population but may also contain other molecules like lipoproteins that may have contributed to the protective effects on hypoxia-exposed H9c2 cells. In any case, however, while the assessment of EVs regarding isolation methods, quantification, and labelling leaves room for misinterpretations and errors, our findings clearly show a positive effect. For the first time, we show that human-derived EV fractions obtained after RIPC from patients undergoing isoflurane anesthesia mediate cell protection against hypoxia, whereas EV fractions from the same patients when obtained before RIPC or from control patients do not.

In summary, our results reveal EVs to be potentially important players in RIPC mediated cell protection and implicitly suggest that RIPC does not only modify EV quantity but also EV quality. Furthermore, propofol impaired this EV mediated protection. Further investigations addressing the cellular origin of EVs, definition of their cargo, and analysis of possible effector mechanisms are necessary, keeping in mind a potential role of EV fractions as a therapeutic tool.

## Supporting information

S1 Dataset (XLSX)

S1 Raw images (PDF)
