# Supplementary figures and images for: Extracellular vesicles isolated from patients undergoing remote ischemic preconditioning decrease hypoxia-evoked apoptosis of cardiomyoblasts after isoflurane but not propofol exposure
Source: PLoS One. 2020 Feb 14;15(2):e0228948. doi: 10.1371/journal.pone.0228948 (PMC7021285; doi:10.1371/journal.pone.0228948)

Figure 3

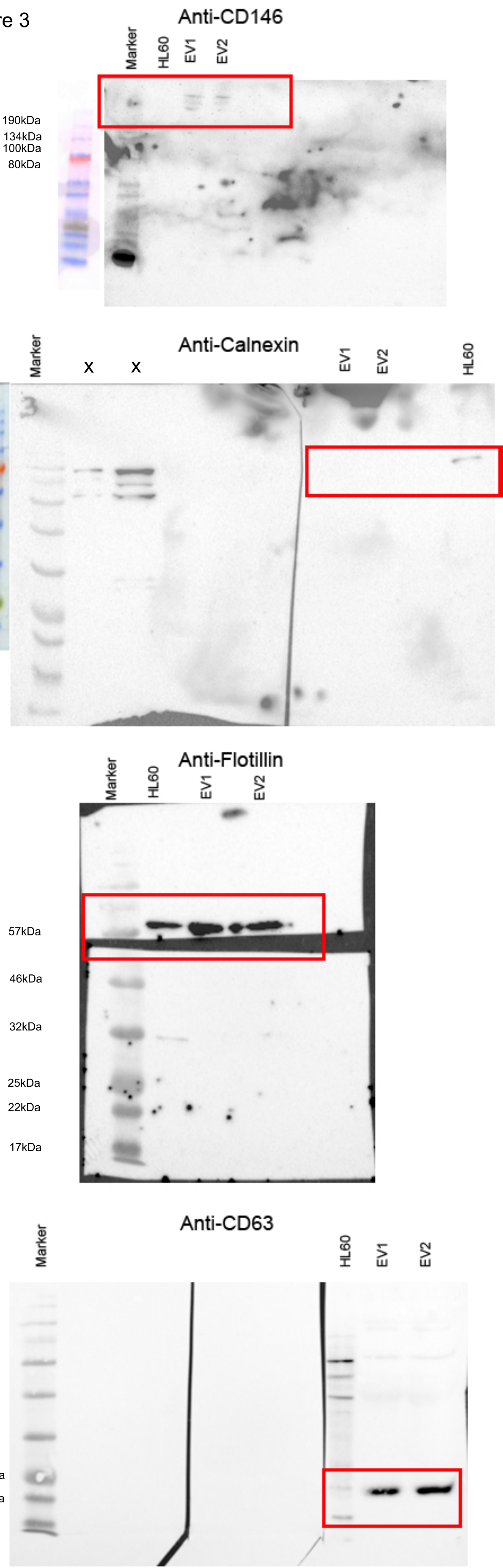

Supplement: S1 Raw images — (PDF) [file pone.0228948.s002.pdf]
